# Long non-coding RNA CRNDE as potential biomarkers facilitate inflammation and apoptosis in alcoholic liver disease

**DOI:** 10.18632/aging.203614

**Published:** 2021-10-11

**Authors:** Yifeng Yan, Liang Ren, Yan Liu, Liang Liu

**Affiliations:** 1 Department of Forensic Medicine, Tongji Medical College, Huazhong University of Science and Technology, Wuhan 430030, China; 2 Department of Forensic Medicine, Wannan Medical College, Wuhu 241002, China

**Keywords:** alcoholic liver disease, lncRNA CRNDE, IL-6, biomarker

## Abstract

Due to persistent inconsistencies in the expression data of alcoholic liver disease (ALD), it is necessary to turn to “pre-laboratory” comprehensive analysis in order to accelerate effective precision medicine and transformation research. We screened pseudogene-derived lncRNA associated with ALD by comparative analysis of 2 independent data sets from GEO. Three lncRNAs (CRNDE, RBMS3-AS3, and LINC01088) were demonstrated to be potentially useful diagnostic markers in ALD. Among them, the expression of CRNDE is up-regulated. Therefore, we focus on CRNDE. Kyoto Encyclopedia of Genes and Genomes pathways analysis revealed higher CRNDE can activate MAPK signaling pathway, apoptosis, wnt signaling pathway, and hematopoietic cell lineage. Next, we established ALD animal model and verified the success of the modeling. The result showed ALD tissues in mice had significantly higher CRNDE levels than normal tissues. Moreover, the increase of IL-6 in the serum of mice in the low-dose group is related to the activation of inflammatory factors after alcohol-induced liver injury. In addition, alcohol can induce apoptosis, and knockdown of CRNDE can reduce apoptosis. Our integrated expression profiling identified CRNDE independently associated with ALD. CRNDE can facilitate inflammation and apoptosis in ALD.

## INTRODUCTION

Alcoholic liver disease (ALD) is the most common type of chronic liver disease in the world [1–3]. ALD can progress from alcoholic fatty liver (AFL) to alcoholic steatohepatitis (ASH), which is characterized by liver inflammation. Chronic ASH eventually leads to fibrosis and cirrhosis, and in some cases hepatocellular carcinoma (HCC). In addition, severe ASH (with or without cirrhosis) can lead to alcoholic hepatitis, which is an acute clinical manifestation of ALD and is associated with liver failure and high mortality [4, 5]. Most people who drink more than 40g a day will develop AFL; however, only some people will develop more serious diseases [6]. Genetic, epigenetic, and non-genetic factors may explain the significant inter-individual differences in the ALD phenotype [7]. The pathogenesis of ALD includes liver steatosis, oxidative stress, acetaldehyde-mediated toxicity, and inflammation induced by cytokines and chemokines [8]. The diagnosis of ALD involves evaluating the patient for signs of alcohol use disorder and advanced liver disease. The degree of AFL and liver fibrosis can be determined by ultrasound, transient elastography, MRI, measurement of serum biomarkers, and liver biopsy histology [9]. Alcoholism through mental and physical intervention is the best treatment for all stages of ALD. In the case of advanced disease such as cirrhosis or HCC, a liver transplant may be required [10]. Therefore, this field needs new biomarkers as diagnostic indicators to be effectively used in individualized treatment.

LncRNA is a type of non-coding RNA with a length greater than 200 nucleotides. Under a variety of stimulus conditions, lncRNA can powerfully increase gene expression and cell differentiation through a variety of molecular mechanisms such as coding gene transcription, epigenetic modification, and post-transcriptional modification, which has become a hot topic in recent years at the transcriptome level [11]. LncRNA can act on protein, and can also act as an endogenous molecular sponge to competitively inhibit the biological activity of miRNA, thereby reducing the transcriptional inhibition of miRNA on its downstream target genes [12]. However, there is still little research on lncRNA involvement in ALD. By using the expression profile, the dysfunctional lncRNA in ALD can be identified. Specific lncRNA has been proposed as a biomarker of the pathogenesis of ALD as a potential therapeutic target.

The purpose of this study was to investigate key lncRNA in ALD by bioinformatics analysis, and explore its possible mechanism. Firstly, we integrate Gene Expression Omnibus (GEO) database to detect the key lncRNA in ALD. Secondly, we perform functional analysis of the target lncRNA and explore its possible mechanism. Thirdly, we successfully established a mice model of alcoholic damage. Based on the successful establishment of a recognized mice model of alcoholic damage, we tested the expression of lncRNA CRNDE in mice liver. In addition, on the basis of successfully establishing a recognized mice model of alcoholic damage, we silenced the content of lncRNA CRNDE in the liver to observe its influence on the occurrence and development of ALD.

## MATERIALS AND METHODS

### Search strategy and data collection, preprocessing, and normalization

GEO database (http://www.ncbi.nlm.nih.gov/geo) was electronically searched to collect eligible expression profiling from inception to April, 2019, with keywords including “Alcoholic Liver Diseases” [MeSH] OR “ALD” [MeSH] OR “Alcoholic Hepatitis” [MeSH] OR “AH” [MeSH] OR “Chronic Alcoholic Hepatitis” [MeSH] AND “Homo sapiens” [MeSH]. Only original experimental studies that screened for different expression profiling between ALD and normal in humans were the first choice for inclusion. Exclusion criteria included: (1) repeated reports by the same institute, hospital; (2) a non-expression gene chip; and (3) a nonwhole-genome chip. Next, we download the probe sequence from GEO and re-annotate the probe using bowtie based on the GENCODE Release 19 annotation for lncRNA. All datasets were normalized individually on the base-2 logarithm by Robust Multi-Array Average (RMA) and Linear Models for Microarray (LIMMA) package and annotated by converting different probe IDs to gene IDs. Finally, the 2 datasets TXT files of all gene lists sorted by logFC were integrated using the RobustRankAggreg (RRA) R package [13].

### Target prediction and functional analysis of lncRNA CRNDE

The presumed targets of integrated-signature lncRNA CRNDE, firstly, the miRNA targets of lncRNA was predicted using the miRcode database (version 11; http://www.mircode.org/) and mRNA targets of miRNAs using miRTarBase (version 7.0), miRDB (version 5.0, http://mirdb.org/) and TargetScan (version 7.2; http://www.targetscan.org/). Unique genes with target sites in 3ʹUTR were incorporated. To assess the prospective functions of the most significant lncRNA CRNDE, we discharged the Kyoto Encyclopedia of Genes and Genomes (KEGG) using the Database for Annotation, Visualization and Integrated Discovery (DAVID). The P value that narrated KEGG pathway enrich the target gene less than 0.05 was defined as the cutoff criterion.

### Preparation of ALD mice model

Adult male SPF BalB/c mice weighing 18-24g were provided by Nanjing Qinglongshan Breeding Farm. Mice were adaptively fed for 2 weeks, freely drinking and eating. 16 mice were randomly divided into dose group I (2 g·Kg^-1^ group), dose group II (4 g·Kg^-1^ group), dose group III (6 g·Kg^-1^ group) and dose group IV (8 g·Kg^-1^ group), each group of 4. After 2 weeks of adaptive feeding, when the mice are in a stable state, Gujing Gongjiu with an alcohol concentration of 45° is selected for gavage experiment on the mice. According to the calculation formula, calculate the amount of alcohol ingested by mice: alcohol intake (ml) = alcohol intake (g) ÷ (alcohol content × 0.8), where alcohol intake (g) = mouse body weight (Kg) × Dose (g·Kg^-1^), and the alcohol content is 0.45, that is, alcohol intake (ml) = mouse weight (Kg) × dose (g·Kg^-1^) ÷ 0.36. The above-mentioned gavage is performed every 3 days, 5 times, and the duration of the gavage is 15 days. Observe and record the behavior, physical signs and death of mice after alcohol gavage. A dose group with a lower mortality rate was selected to prepare for the formal preparation of a mouse alcoholic liver disease model. After 15 days of intragastric administration, the mortality of mice in dose group III and dose group IV was 75% and 100%, respectively. The mortality of mice in dose group III and dose group IV was too high to meet the requirements of the model preparation process. Stability and repeatability requirements. The survival rates of mice in dose group I and dose group II are 100% and 75%, respectively, which can meet the requirements for the preparation of alcoholic liver disease models in mice. Therefore, the gavage doses 2 g·Kg-1 and 4 g·Kg^-1^ corresponding to dose group I and dose group II are selected as the actual gavage dose prepared for this alcoholic liver disease model, where 2 g·Kg^-1^ is The gavage dose of the low-dose group, 4 g·Kg^-1^ was the gavage dose of the high-dose group.

Next, 48 adult male SPF BalB/c mice were randomly divided into a blank control group, a model control group, and an alcoholic liver disease model group. The alcoholic liver disease model group was divided into a low-dose group according to different alcohol doses (2 g·Kg^-1^), high-dose group (4 g·Kg^-1^ body weight), 12 animals per group. The mice in the blank control group were not treated, and the mice in the model control group were given saline. The intake of physiological saline is equivalent to the alcohol intake of the middle-dose group, that is, the intake of physiological saline in the model control group (V health, ml) = 11.11 × mouse body weight (Kg). The different dose groups were given the corresponding alcohol intake of liquor, that is, the alcohol intake of the low-dose group (V low, ml)=5.55×the weight of the mouse (Kg), and the alcohol intake of the high-dose group (V, ml) = 11.11 × mouse body weight (Kg).

### Detection of serum biochemical indicators in mice

According to the peak concentration time of ethanol absorption in the body and the peak concentration time of some biochemical indicators, 9 hours after the completion of the modeling, the mice were taken from the eyeballs and blood was collected, and the blood was collected in an EP tube. The blood sample in the EP tube was balanced and placed in a high-speed refrigerated centrifuge., 1200g, 4° C centrifugation for 10min, after centrifugation, take the supernatant (serum) and store it in the -80° C refrigerator. Using Hitachi 7600 automatic biochemical analyzer and Biyuntian Biotechnology Co., Ltd. detection kit to determine serum biochemical indicators, including aspartate aminotransferase (AST), alanine aminotransferase (ALT), total bilirubin (TBIL), and direct bilirubin (DBIL).

### Tissue collection

After the mice was sacrificed, the mice’s chest and abdomen were opened, and the mice’s liver, brain, heart, lung, spleen, pancreas, and kidney were quickly taken out and placed in a numbered 4ml EP tube, placed on dry ice for quick freezing and then placed in the refrigerator at -80° C. Take part of the liver tissue and place it in a 10% paraformaldehyde solution for fixation for 24 hours. The remaining organs are stored in a refrigerator at -80° C for inspection. Take out the liver tissue fixed with 10% paraformaldehyde solution, put it face down into the embedding box, dehydrate it on a dehydrator and then use xylene for transparency. Finally, the transparent brain tissue is immersed in wax and wrapped. After hematoxylin-eosin (HE) staining of liver tissues of each group of mice, the Leica microscope was used to observe and collect images.

### CBA method to detect mice serum cytokines

Take blood from mouse eyeballs and place them in 2.5ml EP tube at 1500rpm×15min. After centrifugation, the upper layer is serum, and the lower layer is blood clot. Take the upper layer of serum into a 1.5ml EP tube and place it in a refrigerator at -80° C. Starting from the highest concentration standard, add 300μl multiples of diluent one by one, and dilute the standard gradually. Take 300μL of the solution from the highest concentration standard tube and add it to the 1:2 tube, pipette to mix, and then take 300μl from the 1:2 tube to the 1:4 tube, and so on, until the 1:256 tube. FCM software was used to analyze and calculate the expression of IL-6, IL-10, MCP-1, TNF, IFN-γ, and IL-12p70 on each group of cells.

### Determination of the apoptosis rate of mice hepatocytes

The hepatocytes in the logarithmic growth phase were processed according to the aforementioned grouping method, and washed with PBS. Then add an appropriate amount of trypsin to digest the cells, discard the trypsin, add the culture medium to resuspend the cells and count. Use flow cytometry to detect cell apoptosis rate.

### Statistical analysis

Data are all expressed by (mean ± SEM); homogeneity of variance data, one-way analysis of variance (one-way ANOVA) is used for multiple comparisons between groups, Student’s-t test is used for pairwise comparisons between groups. All data were statistically analyzed by SPSS 17.0 (SPSS, Chicago, IL) software. P<0.05 indicates that the difference is statistically significant. The test level α=0.05, P<0.05 indicates that the difference is statistically significant.

## RESULTS

### Integrated analysis of lncRNA CRNDE in 2 datasets

**Figure 1:**
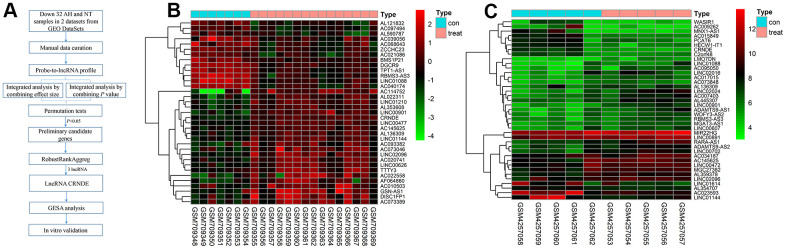
**Differentially lncRNAs in alcoholic liver disease.** (**A**) The flowchart of the integrated analysis and functional validation. *In silico* bioinformatics data analysis pipeline consists of curation of 2 publicly available datasets, data preprocessing, and integrated analysis, dataset validation, GSEA, and *in vitro* functional validation. (**B**) Heatmap of differentially expressed lncRNAs in GSE28619 dataset; each column represents a sample and each row represents a specific lncRNA. |log2FC| >1 and adjusted P<0.05. Red indicates upregulation and green indicates downregulation of expression. (**C**) Heatmap of differentially expressed lncRNAs in GSE143318 dataset; each column represents a sample and each row represents a specific lncRNA. |log2FC| >1 and adjusted P<0.05. Red indicates upregulation and green indicates downregulation of expression.

**Figure 2:**
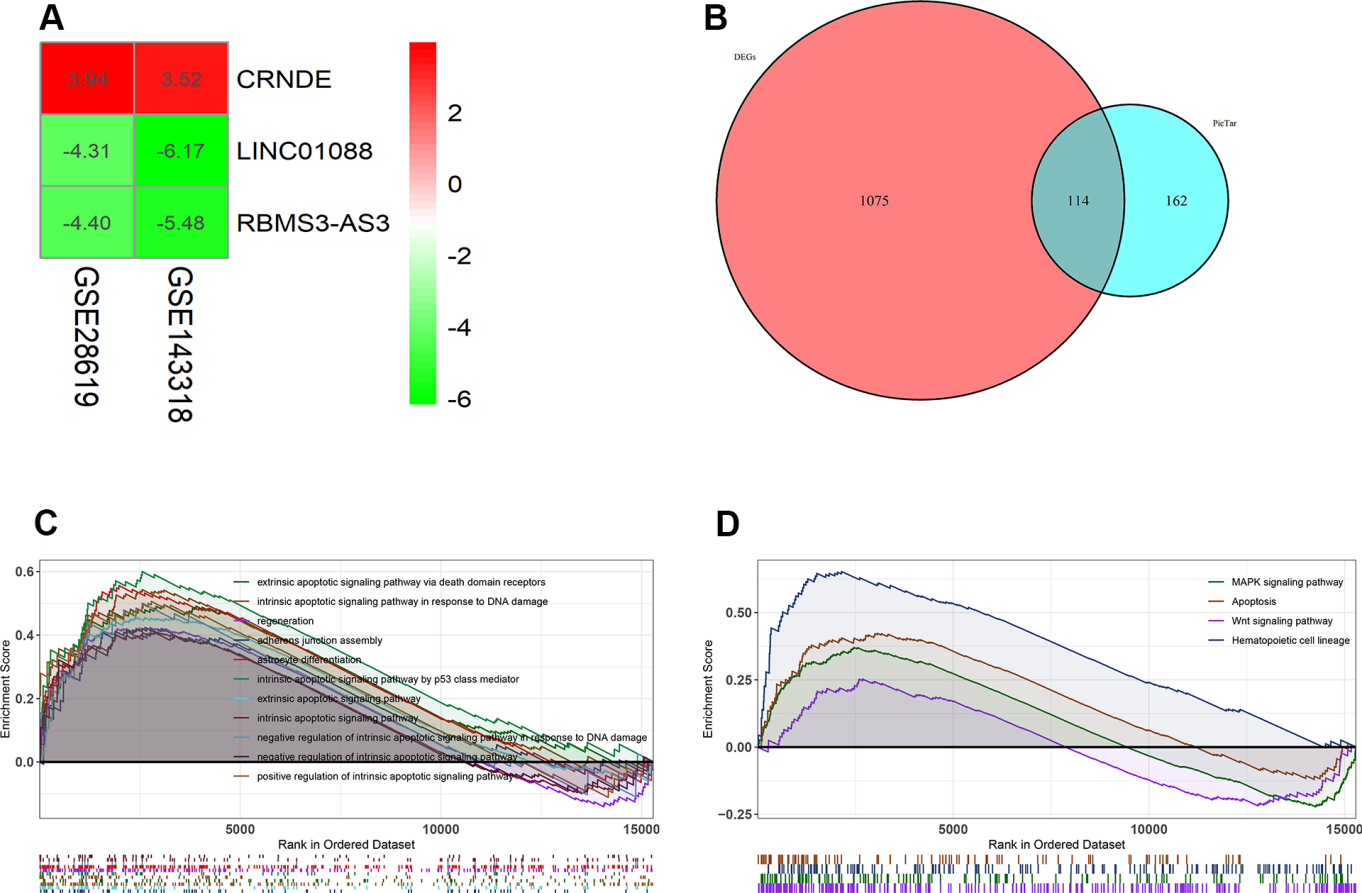
**Lnc CRNDE may serve as significant markers in alcoholic liver disease.** (**A**) Log FC heatmap of each expression microarray, the abscissa represent the GEO IDs, the ordinate represents the gene name, the red represents log FC > 0, the green represents log FC < 0 and the value in the box represents the log FC value. (**B**) Venn diagrams of predicted lnc CRNDE targets genes and differentially expressed genes in GSE28619 dataset. (**C**) Biological processes enriched in alcoholic liver disease. (**D**) KEGG pathways enriched in alcoholic liver disease.

We located and manually curated 359 published and publicly available ALD datasets. We identified 2 datasets (GSE28619 [14] and GSE143318 [15]) meet the criteria including 20 ALD samples and 12 normal. The study selection flow chart for this integrated analysis is shown in Figure 1A. Using p <0.05 and [logFC]>1 as cut-off criterion, a total of 39 lncRNAs were significantly deregulated in GSE143318 datasets, and 35 in GSE28619 datasets (Figure 1B, 1C). After merging the datasets, 3 differential lncRNAs were identified including 2 downregulated lncRNAs (BMS3-AS3 and LINC01088) and 1 upregulated lncRNA (lncRNA CRNDE) (Figure 2A). Therefore, we focus on lncRNA CRNDE.

Next, in order to predict the possible potential functions of lncRNA CRNDE, we first predicted the target genes of lncRNA CRNDE, and used these target genes to intersect the difference genes of GSE28619. A total of 114 genes were found (Figure 2B). In addition, we use GSEA to analyze the function of 114 genes, and GSEA GO analysis revealed lncRNA CRNDE is significantly involved in the extrinsic apoptotic signaling pathway via death domain receptors, intrinsic apoptotic signaling pathway in response to DNA damage, regeneration, and regulation of adherens junction organization (Figure 2C). GSEA KEGG pathways analysis revealed lncRNA CRNDE is significantly involved in the MAPK signaling pathway, apoptosis, wnt signaling pathway and hematopoietic cell lineage (Figure 2D). In summary, lncRNA CRNDE may play a important role in the apoptosis pathway.

### Preparation of ALD mice model

In order to verify the function of lncRNA *in vitro*, we have established an ALD model. As shown in Table 1, the weight of mice in the control group and the model group showed an upward trend. The weight of the mice in the low-dose group and the high-dose group was lower than that of the blank control and the model control group, and the weight of the mice in the low-dose and high-dose groups changed steadily, especially the high-dose group. The survival rate of mice in the blank control group and the model control group was 100%. The survival rates of the mice in the low-dose group and the high-dose group were 83.33% and 58.33%, respectively. The survival rate of the mice in the high-dose group was obvious lower in the lower-dose group (Table 2). Next, we detect the serum biochemical indicators in mice. Compared with the blank control group, the serum TP, GLOB, ALT and AST of the mice in the high-dose group were all increased (P<0.05). The serum ALB of the mice in the high-dose group was lower than that of the blank control group (P<0.05). Compared with the control group, mouse serum TBIL, DBIL and GGT did not change significantly (Table 3).

**Table 1:** Changes of body weight of mice in each group before and after gavage.

	**Blank control**	**Model control**	**Low dose**	**High dose**
**1d**	19.50±1.09	19.83±1.19	19.33±1.67	20.75±1.82
**43d**	29.18±2.27	28.75±1.48	29.60±1.65	27.00±1.63
***t* value**	12.85	16.21	14.45	7.5
***P* value**	<.001	<.001	<.001	<.001

**Table 2:** Survival and death of mice during the preparation of alcoholic liver disease model.

	**Blank control**	**Model control**	**Low dose**	**High dose**
**N**	12	12	12	12
**Survival**	12	12	10	7
**Number of deaths**	0	0	2	5
**Survival rate**	100%	100%	83.33%	58.33%

**Table 3:** The expression level of biochemical indexes in serum of mice.

	**Blank control**	**Model control**	**Low dose**	**High dose**	***P* value**
**TP(g/L)**	63.8±1.98	61.3±1.84	62.35±2.20	68.43±2.46	*0.014*
**ALB(g/L)**	39.05±0.64	34.40±1.70	33.98±2.35	34.43±3.05	0.169
**GLOB(g/L)**	27.50±0.71	26.90±0.14	28.34±1.48	34.00±2.79	*0.045*
**TBIL(μmol/L)**	-0.50±0.20	-0.50±0.06	-0.45±0.39	-0.32±0.31	0.882
**DBIL(μmol/L)**	-1.23±0.18	-0.97±0.49	-0.64±0.44	-0.93±0.16	0.303
**ALT(U/L)**	24.00±9.90	24.50±2.12	32.25±2.36	39.25±4.57	*0.019*
**AST(U/L)**	61.00±1.41	71.50±6.36	88.25±1.26	110±20.15	*0.007*
**GGT(U/L)**	5.50±0.71	4.00±1.41	4.25±1.50	3.50±1.73	0.532

P value less than 0.05 is considered as significance with italic fonts.

**Figure 3:**
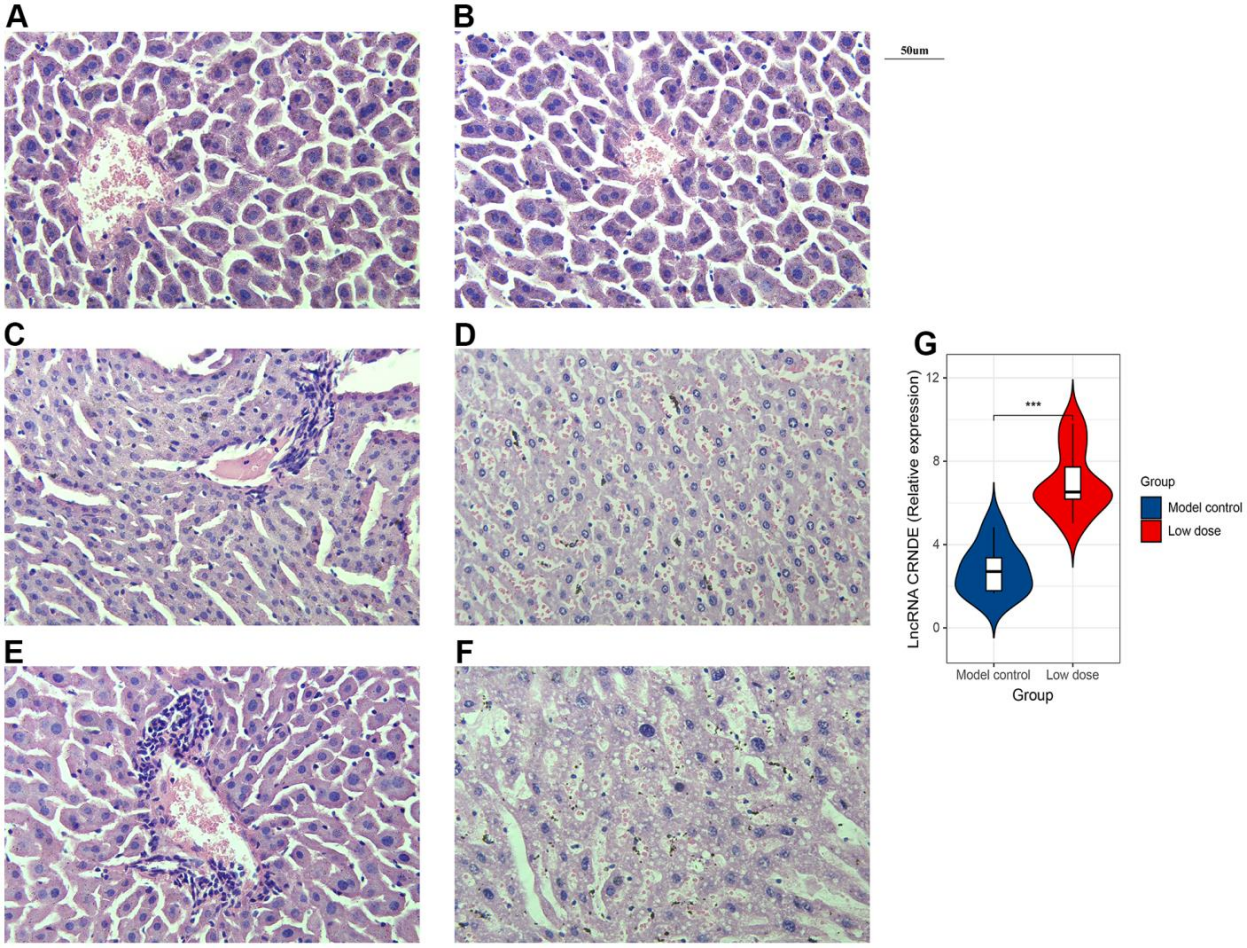
**Successfully constructed alcoholic liver disease mice model.** (**A**) HE staining image of blank group. (**B**) HE staining image of model control. (**C**, **D**). HE staining image of low dose group. (**E**, **F**) HE staining image of high dose group. (**G**) qRT-PCR was performed to detect relative lnc CRNDE expression in low dose group and model control group. *P < 0.05, **P < 0.01, ***P < 0.001.

Next, in order to verify whether alcohol can affect the liver structure, we use HE staining. The results of the blank group and the model showed that the structure of the liver lobules was clearly distinguishable, the liver cords were arranged radially, the liver cells were present, the nuclei were large and round, regular in size, blue stained, cytoplasm powder stained, no steatosis or inflammatory cell infiltration (Figure 3A, 3B). The results of the low-dose group showed that the structure of the liver lobules was discernible, the hepatic cords were arranged irregularly, the erythrocytes in the sinusoids were stagnated, and a small amount of inflammatory cell infiltration around the blood vessels (Figure 3C). Liver cells are swollen, with nuclei of varying sizes, a small amount of nuclei disappeared, cytoplasm is loose, lightly stained and homogeneous, and vacuoles appear in the cytoplasm of some liver cells with varying sizes (Figure 3D). Moreover, the results of the high-dose group showed that the structure of the liver lobules is still indistinguishable, the hepatic cords are arranged disorderly, the red blood cells in the sinusoids are stagnated, and a large number of inflammatory cells infiltrate around the blood vessels (Figure 3E). The cytoplasm of hepatocytes is loose and lightly stained, the nucleus staining varies in depth and size, and some of the nuclei of hepatocytes disappear; the cytoplasm is lightly stained, and vacuoles appear in the cytoplasm of most hepatocytes with varying sizes (Figure 3F). In conclusion, the mice ALD model was validated by serum biochemical test and HE staining. The results showed that the animal model of chronic alcohol infusion in mice was successfully established, for the next experimental study of the function of lncRNA CRNDE and other animal models to lay a solid foundation.

### Increased lncRNA CRNDE expression in ALD mice

**Figure 4:**
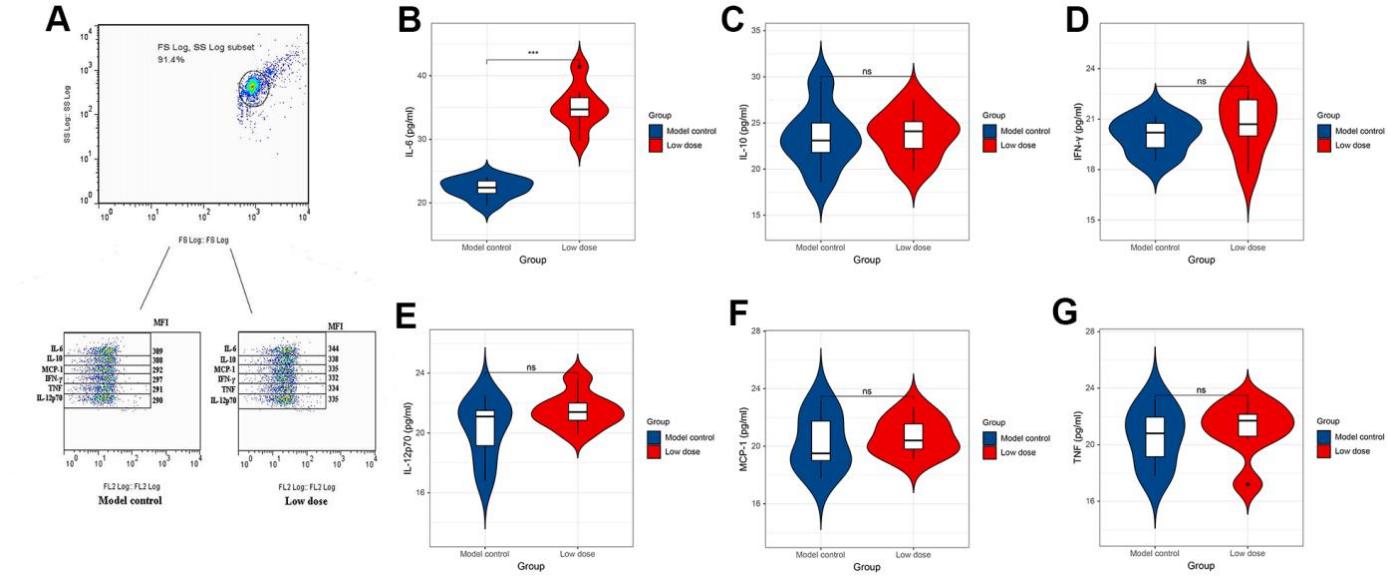
**CBA method to detect changes in the expression level of cytokines in serum of mice.** (**A**) Expression level of cytokines in serum of mice. (**B**–**G**) Expression levels of IL-6, IL-10, MCP-1, TNF, IFN-γ and IL-12p70 in serum of mice. *P < 0.05, **P < 0.01, ***P < 0.001.

To investigate the correlation between lncRNA CRNDE and ALD, we performed qRT-PCR to detect relative lncRNA CRNDE expression ALD mice, and found ALD tissues in mice had significantly higher lncRNA CRNDE levels than normal tissues (Figure 3G). Due to inflammatory mediators play a vital role in the occurrence and development of alcoholic liver disease, therefore, we use the CBA method to detect changes in the expression of mice serum cytokines (Figure 4A). The results showed that compared with the control group, the expression of IL-6 in the low-dose group was significantly increased (Figure 4B), while other inflammatory mediators such as IL-10, MCP-1, IFN-γ, TNF, IL-12p70 did not change significantly (Figure 4C–4G).

### Increased lncRNA CRNDE facilitates apoptosis in ALD

**Figure 5:**
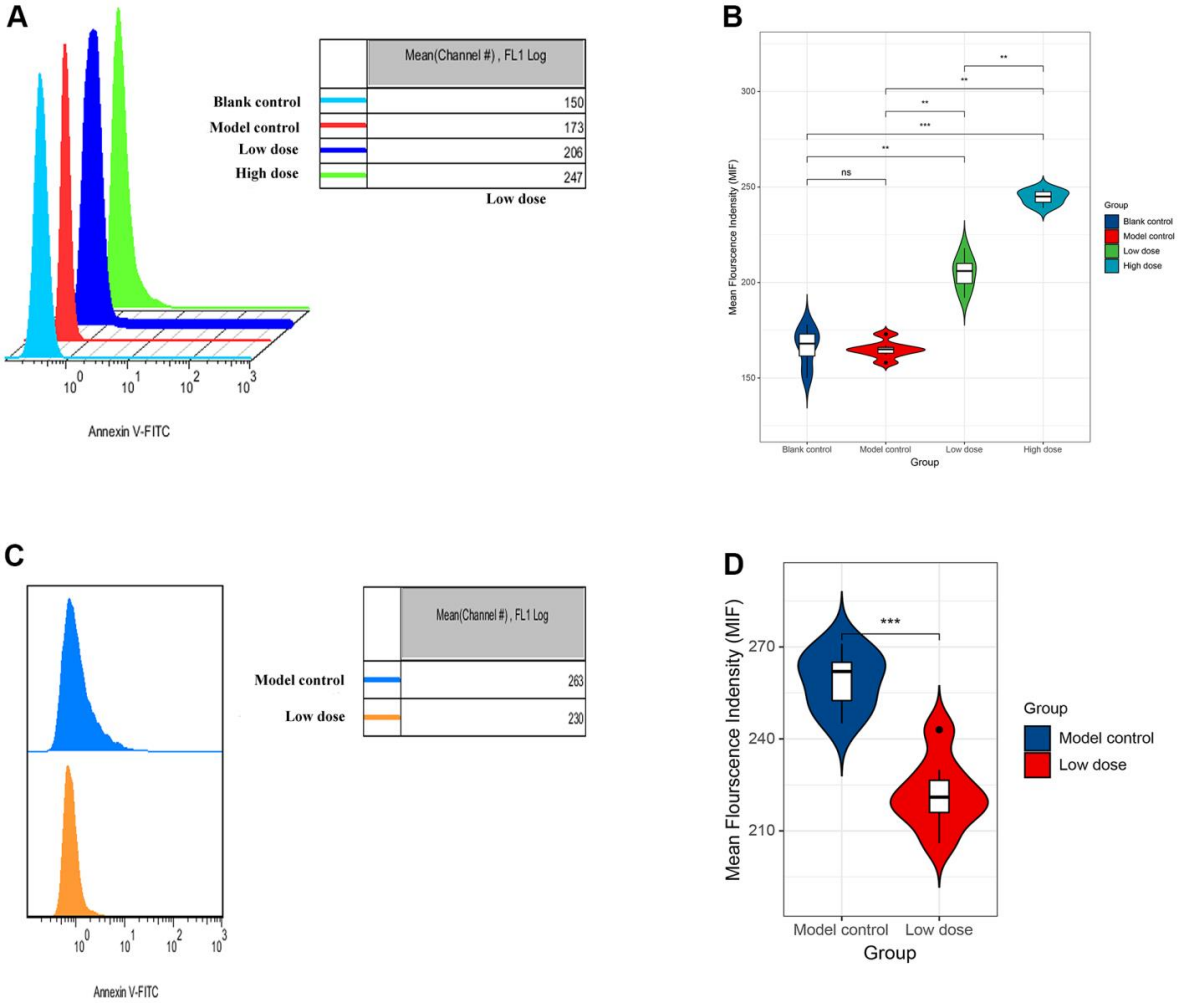
**Increased lncRNA CRNDE facilitates apoptosis in alcoholic liver disease mice.** (**A**) Flow cytometry was used to detect the apoptosis rate of liver cells in mice. (**B**) The statistic figure of apoptosis rate of mice hepatocytes detected by flow cytometry. (**C**) Changes of apoptosis rate of liver cells in mice after interference with lncRNA CRNDE. (**D**) The statistic figure of apoptosis rate of mice hepatocytes detected by flow cytometry after interference with lncRNA CRNDE. *P < 0.05, **P < 0.01, ***P < 0.001.

In order to explore the possible mechanism of lncRNA CRNDE in ALD, we used flow cytometry to detect the apoptosis rate of mice hepatocytes. Data revealed that compared with the model control group, the apoptosis rate of mice hepatocytes was significantly increased. Moreover, with the increase of alcohol concentration, the higher the apoptotic rate of mice liver cells (Figure 5A, 5B). Next, mice receiving saline were injected with the same amount of empty virus (Sh-RNA group) and low-dose alcoholic were injected with the same amount of siRNA-CRNDE, and then perform flow cytometry detection on the model control group and low-dose group. We found that compared with the model control group, the mouse liver cell apoptosis rate in the low-dose group was significantly reduced (Figure 5C, 5D). In conclusion, lncRNA CRNDE may affect ALD through apoptotic pathway.

## DISCUSSION

In this study, we use a unified cross-platform standardized genetic analysis method. This method ignores the unavailability of raw data and can be used to identify lncRNA expression in tissues. This method is widely used in cancer. Our current research supports the effectiveness of this comprehensive strategy in ALD, and has identified 3 ALD-related lncRNAs. Through bioinformatics analysis, we found that 3 lncRNAs are related to ALD, and found that the main function of lncRNA CRNDE may be related to the apoptosis pathway. Next, we successfully established the ALD model and verified its success. At the same time, we found that the expression of lncRNA CRNDE in the ALD model was elevated, indicating that lncRNA CRNDE can be an effective diagnostic marker in the clinical environment. Finally, by interfering with lncRNA CRNDE, we found that the apoptotic rate of mice decreased, indicating that lncRNA CRNDE may affect ALD through the apoptotic pathway. As far as we know, this is the first time that multiple chips have been used to analyze that lncRNA CRNDE may be a marker of ALD. Successfully verified the pathogenic role of lncRNA CRNDE in ALD *in vitro*. Our research results indicate that lncRNA CRNDE may be a potential molecular target that interferes with the occurrence and development of ALD, thereby providing new insights into the pathogenesis of ALD.

After the body consumes alcohol, 90% of the alcohol is metabolized by the liver [16]. Long-term high-dose alcohol intake can cause liver damage to varying degrees, such as liver steatosis, hepatitis, cirrhosis, and even hepatocellular carcinoma [17]. Studying the mechanism of alcoholic liver injury by replicating the animal model of alcoholic liver disease and finding more effective therapeutic drugs has become an important problem that urgently needs to be faced and solved. Acute alcohol intragastric model and chronic alcohol feeding model are commonly used in the preparation of acute alcoholic liver injury models. Their advantages are simple and easy to operate, and the modeling period is short. Their disadvantages are that they can only cause slight liver injury, which cannot meet the requirements of some experimental models [18, 19]. Chronic alcoholic liver disease and liver fibrosis models can cause more severe alcoholic liver fibrosis, but the high mortality of mice during the modeling process limits the administration of more alcohol and the more severe induction of alcoholic liver injury in mice [20]. In conclusion, no effective method of alcohol alone to cause chronic alcoholic liver cancer in animal models has been found. The model of alcoholic liver disease in mice was established by using modified chronic alcohol gavage. First of all, the appropriate dose range of alcohol was selected in the pre-experiment of the mouse model of alcoholic liver disease. The pre-experiment results showed that the suitable dose range of 45 degree liquor (Gujing Gong liquor, Bozhou) was 0~4 g· kg^-1^, and the survival rate of mice was low when the dose was 6~8 g· kg^-1^.

Albumin (ALB) and globulin (GLOB) are common serum proteins, which are found in almost all animals and plants. Albumin and globulin play an immune role. When the body is stimulated by external factors, its serum expression level changes accordingly. In chronic hepatitis, cirrhosis, especially liver cancer, albumin decreases while globulin increases. However, there was no significant change in acute hepatitis [21, 22]. ALT and AST two kinds of transaminases mainly exist in the liver. When liver cells are damaged or necrotic, this enzyme will be increased in the blood [23]. In this experiment, the expression levels of ALT, AST, TP and GLOB in serum of mice in high-dose group were increased compared with blank control group (P < 0.05), while ALB was significantly decreased compared with blank control group (P < 0.05). The results of HE staining under microscope showed that the low-dose group had hepatocyte cytoplasmic loosening and perivascular inflammation. The high-dose group had hepatocyte steatosis, mild hepatocyte necrosis, and significant inflammatory response. The ALD model of mice was verified by serum biochemical detection and HE staining. The verification results showed that this experiment successfully established the mouse model of chronic alcohol in gastric administration, which laid a solid foundation for the following experimental studies, including the screening of lncRNAs and related functional studies.

LncRNA plays a key role in many human diseases, among which liver disease is a very important aspect [24]. LncRNA expression varies significantly in hepatocellular carcinoma and various types of hepatitis [25]. The four lncRNAs HOTAIR1, MALAT1, MEG3 and H19 have been proved to be highly correlated with primary liver cancer [26–29]. Not only in liver cancer, but also in hepatitis B, the overexpression of lncRNA-HOTAIR has recently been found to have regular interaction with HBV and HCC [30]. In cirrhosis (such as PBC), the gene GAS5 was screened out, which was preliminarily confirmed to be a biological marker of primary biliary cirrhosis and has a broad clinical application prospect [31]. Drug-induced liver injury is also a common type of liver injury in clinic. Using chip detection technology, researchers found that lncRNA expression also changed after the occurrence of such liver injury. After acetaminophen induced liver injury in mice, LOC100861856, GM19933, A130040M12RIK were found to be upregulated, while C730036E19RIK, 13000015D01RIK, GM19894 were down-regulated. In mouse liver ischemia reperfusion, many lncRNAs are up-regulated, especially AK139328, which shows its potential application value [32]. More importantly, lncRNAs are also overexpressed in alcoholic liver injury. In the mouse animal model, H19 expression was up-regulated and MEG3 expression was down-regulated in liver [33]. All the above research results showed that lncRNA expressed varying degrees of research value and profound clinical diagnosis and treatment potential value in many types of liver diseases, such as hepatocellular carcinoma, viral hepatitis, cirrhosis, hepatic ischemia reperfusion, and alcoholic liver disease.

LncRNA CRNDE is a long non-coding RNA specifically expressed in time and tissue, located at HCG_1815491 gene locus on chromosome 16 [34, 35]. The expression of lncRNA CRNDE gene was up-regulated in both HCC tissues and HCC cell lines, and the expression of CRNDE was correlated with the clinicopathological characteristics of HCC, such as clinical stage and lymph node metastasis [36]. Esposti et al. analyzed the expression of lncRNA CRNDE in 10 liver cancer tissues by RNA sequencing technology, and found that the expression of lncRNA CRNDE was upregulated, increasing by 1.54 times [37]. LncRNA CRNDE plays an important role in promoting the cell viability, proliferation and angiogenesis of hepatoblastoma *in vitro*, as well as tumor growth and angiogenesis *in vivo*. CRNDE inhibited miR-384 and regulated the expression of NF-κB and p-Akt to promote the proliferation, migration and invasion of HCC cells. In addition, CRNDE leads to angiogenesis through activation of the mTOR signaling pathway [38].

In this study, we screened pseudogene-derived lncRNA associated with ALD by comparative analysis of 2 independent data sets from GEO, and found lncRNA CRNDE may be an effective gene target for ALD. Next, KEGG pathways analysis revealed lncRNA CRNDE is significantly involved in the MAPK signaling pathway, apoptosis, wnt signaling pathway and hematopoietic cell lineage. We established ALD animal model and verified the success of the modeling, and found ALD tissues in mice had significantly higher lncRNA CRNDE levels than normal tissues. In addition, alcohol can induce apoptosis, and interfere with lncRNA CRNDE can reduce apoptosis.

However, there are some limitations in our study. Firstly, although an earnest endeavor was made to recruit as many ALD patients from GEO database as possible to validate lncRNA CRNDE in ALD, this study was still only including 20 ALD samples and 12 normal. Secondly, although we identified and validated potential biomarker, we did not experimentally evaluate their efficacy. There is a need for multi-center prospective studies to evaluate their application. Finally, we did not obtain sufficient clinical information for the subjects herein; therefore, the clinical value of our findings needs further verification.

Our experiment is the first to find that lncRNA CRNDE independently associated with ALD by integrated bioinformatics, and found ALD tissues in mice had significantly higher lncRNA CRNDE levels than normal tissues. Moreover, the increase of IL-6 in the serum of mice in the low-dose group is related to the activation of inflammatory factors after alcohol-induced liver injury. In addition, alcohol can induce apoptosis, and interfere with lncRNA CRNDE can reduce apoptosis. In conclusion, our research results indicate that lncRNA CRNDE may be a potential molecular target that interferes with the occurrence and development of ALD, thereby providing new insights into the pathogenesis of ALD.
